# DeepSeqPan, a novel deep convolutional neural network model for pan-specific class I HLA-peptide binding affinity prediction

**DOI:** 10.1038/s41598-018-37214-1

**Published:** 2019-01-28

**Authors:** Zhonghao Liu, Yuxin Cui, Zheng Xiong, Alierza Nasiri, Ansi Zhang, Jianjun Hu

**Affiliations:** 1 Department of Computer Science and Engineering, University of South Carolina, 29201 Columbia, SC United States; 2 School of Mechanical Engineering, Guizhou University, 50033 Guiyang, Guizhou China

## Abstract

Interactions between human leukocyte antigens (HLAs) and peptides play a critical role in the human immune system. Accurate computational prediction of HLA-binding peptides can be used for peptide drug discovery. Currently, the best prediction algorithms are neural network-based pan-specific models, which take advantage of the large amount of data across HLA alleles. However, current pan-specific models are all based on the pseudo sequence encoding for modeling the binding context, which is based on 34 positions identified from the HLA protein-peptide bound structures in early works. In this work, we proposed a novel deep convolutional neural network model (DCNN) for HLA-peptide binding prediction, in which the encoding of the HLA sequence and the binding context are both learned by the network itself without requiring the HLA-peptide bound structure information. Our DCNN model is also characterized by its binding context extraction layer and dual outputs with both binding affinity output and binding probability outputs. Evaluation on public benchmark datasets shows that our DeepSeqPan model without HLA structural information in training achieves state-of-the-art performance on a large number of HLA alleles with good generalization capability. Since our model only needs raw sequences from the HLA-peptide binding pairs, it can be applied to binding predictions of HLAs without structure information and can also be applied to other protein binding problems such as protein-DNA and protein-RNA bindings. The implementation code and trained models are freely available at https://github.com/pcpLiu/DeepSeqPan.

## Introduction

Human leukocyte antigens (HLAs) are major histocompatibility complex (MHC) proteins located on the cell surface in human. HLAs play a critical role helping our immune system recognizing pathogens by binding to peptide fragments derived from pathogens and exposing them on the cell surface for recognition by appropriate T cells. Study of the binding mechanism between peptides and HLAs can help improve our understanding of human immune system and boost the development of protein-based vaccines and drugs^1,2^. Out of all classes of HLAs, we are interested in two major classes: class I and II. Class-I HLAs bind to peptides inside the cell while class-II HLAs bind to peptides from extracellular proteins that are brought inside the cell.

A big challenge of determining peptides binding to HLAs is the high polymorphism of HLA genes. As of March 2018, there are more than 17000 HLA alleles deposited in the IMGT/HLA database. Experimentally testing the binding between peptides and different types of HLAs is costly and time-consuming. As a result, computational methods have been proposed to address this problem as more and more *in vitro* binding affinities data are published in databases such as IEDB^3^, SYEPEITHI^4^ and MHCBN^5^.

Generally, current computational methods for peptide-HLA binding affinity prediction can be grouped into two categories: allele-specific and pan-specific models^2,6–13^. Allele-specific models are trained with only the binding peptides tested on a specific allele and a separate allele-specific binding affinity prediction model is needed for each HLA allele. NetMHC^1^ and SMM^7^ are the top allele-specific MHC binding prediction models. These models have the advantage of good performance when sufficient number of training peptide samples are available. However, due to the high polymorphism, for many HLA alleles, there are no or just a few experimentally determined binding affinity data. To address this data scarcity issue, pan-specific methods have been proposed and have achieved significant improvement in terms of prediction performance^14^. In these models, binding peptides of different alleles are all combined to train a single prediction model for all HLA alleles. Typically, a pan-specific model uses binding affinity data from multiple alleles for training and could predict peptide binding affinity for the alleles that may have or have not appeared in the training data. The key idea behind pan-specific models is that besides encoding the peptide in a proper way for the prediction model, the peptide-HLA binding context/environment is also represented so that the machine learning models could be trained on all available peptide-HLA binding samples^14^. In other words, both the peptide and the HLA protein are encoded as input to the pan-specific models to train the prediction models. So far, a number of pan-specific models have been proposed for both HLA class I and class II alleles^14^. Among them, NetMHCPan, PickPocket and Kim *et al*.’s work are recently proposed pan-specific HLA-peptide binding prediction models trained on the large amount of HLA class I binding affinity data.

**Figure 1:**
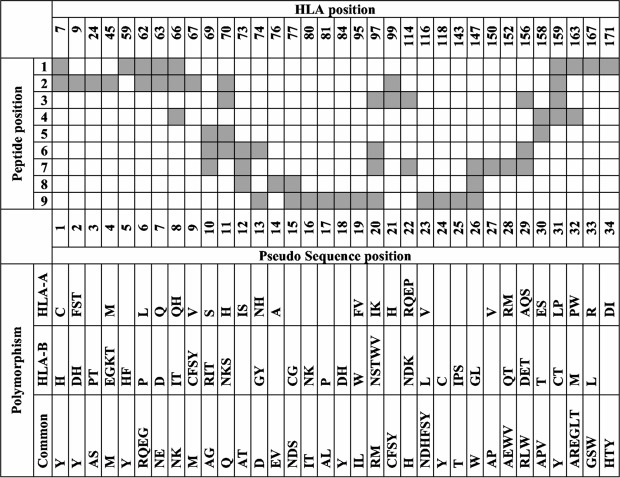
Interaction map of the HLA pseudo sequence in NetMHCPan. Reproduced from original paper.

NetMHCPan is the first pan-specific binding affinity prediction algorithm that takes a large number of peptide-HLA binding samples of different HLA alleles for model training and obtained state-of-the-art performance^6^. NetMHCPan proposed a novel pseudo sequence encoding method to represent the binding context, in which an HLA sequence is reduced to a pseudo amino acid sequence of length 34. Each amino acid in this pseudo sequence is selected if it is in contact with the peptide within 4.0 $\dot{A}$ (as shown in Fig. 1). The interaction map in Fig. 1 is extracted based on a representative set of HLA structures with nonamer peptides. Actually, this extracted 34-length pseudo sequence is a list of location indexes of amino acids in the HLA sequence. For a given HLA sequence, only 34 corresponding residues are encoded as input. In NetMHCPan, an HLA-peptide binding sample is represented as a 43-length residue sequence (9 from the peptide and 34 from the HLA). This sequence is then encoded in three different ways: one-hot encoding, BLOSUM50, or a mixture of both. The encoded input is then used to train multiple feedforward neural networks with 22 to 86 hidden neurons. Then the network with the highest prediction performance (lowest square error) on the test set was selected as the final prediction model^6^. This pseudo sequence encoding approach for pan-specific modeling has also been used in PickPocket^11^ and Kim’s algorithm^12^, but with different machine learning algorithms for model training. In PickPocket, position-specific scoring matrices (PSSMs) are first derived from peptides data. Then extract the position-specific vectors from the PSSMs in association with pseudo-sequence to construct a pocket library. Each pocket library entry is characterized by nine pairs, where each pair consists of a list of pocket amino acids and a specificity vector.

Deep convolutional neural networks (DCNN) are powerful deep learning models and have been successfully applied in many bioinformatics problems such as DNA-binding prediction and CpG island binding prediction^15,16^. This technique has also been applied to peptide-MHC binding prediction^12,17,18^. Among them, Kim *et al*. proposed a pan-specific DCNN model for peptide-MHC class I binding prediction^12^, in which the peptide-binding context is encoded using NetMHCPan’s contact residue sequence method and the DCNN model is trained as a 26-layer classifier. Recently, John *et al*.^19^ introduced a preliminary work which does not involve with pseudo sequence encoding for MHC-peptide binding. It used convolutional layers and trainable embedding to encode allele sequences. However, its network structure and encoding details are not very clear since it does not give the implementation code.

With this background, the pseudo sequence encoding proposed in NetMHCPan is currently the dominating binding context encoding method in pan-specific peptide-HLA class I binding prediction, which has achieved state-of-the-art performance in the public benchmark study^20^. This encoding method has its potential limitations: 1) its interaction map extraction step relies on available MHC-peptide bound complex structures, which may not be always available, especially considering the high polymorphism of HLA proteins; 2) the 34 contact residues of the encoding is empirical and only covers part of the whole HLA sequence. We recognize that current pseudo-sequence based methods do not require MHC-peptide bound complex structures to train or predict thanks to the extreme conservation of the MHC-I structure (even among different species). The 34 positions used in pseudo-sequence coding remain the same for every one of such molecules discovered so far. So, after figuring out the 34 positions of the HLA protein sequence critical to binding, no structural information is needed for new MHC-I proteins if these positions are conserved.

**Figure 2:**
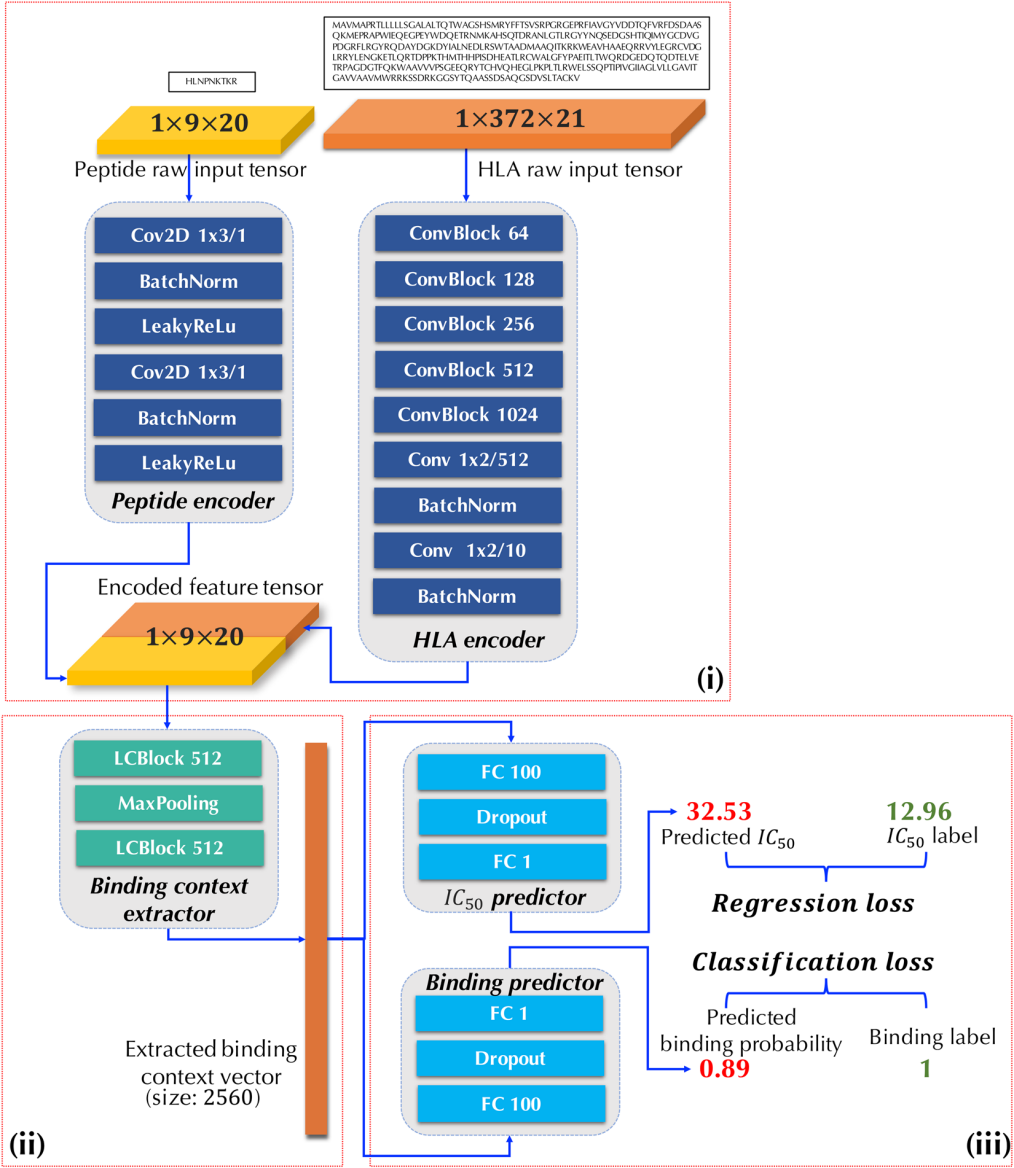
DeepSeqPan Network Structure. (i) Peptide and HLA encoders. (ii) Binding context extractor. (iii) Affinity and binding predictors.

In this paper, we propose DeepSeqPan (Fig. 2), a deep neural network trained on pairs of one-hot encoded raw peptides and HLA sequences, which make it possible to train pan-specific HLA-peptide binding prediction model without the three-dimensional structural data during training stage. Evaluation on the independent IEDB benchmark datasets showed that our proposed model achieved state-of-the-art performance on many HLA alleles.

Our contributions can be summarized as follows:

1. We proposed a novel DCNN architecture for pan-specific HLA binding prediction, in which the peptide-HLA binding context is learned by the network itself. Our DeepSeqPan does not rely on any direct or derived structure information during training stage.
2. Designed as a multi-task learning problem, our DCNN predicts both *IC*_50_ values and binding probabilities at the same time.
3. Our pan-specific prediction model has demonstrated high generalization capability across alleles. The blind testing shows that the network could learn the basic cross allele features when trained with reasonable number of samples.

## Method

### Dataset

The training dataset BD2013 is downloaded from the widely used IEDB^3^ database (http://tools.iedb.org/main/datasets). All training samples are labeled with *IC*_50_ binding affinity values. The testing dataset is downloaded from IEDB’s weekly benchmark dataset ranging from 2014-03-21 to 2018-01-26 (http://tools.iedb.org/auto_bench/mhci/weekly). To address the concern that duplicate peptides may exist in both the training and testing data downloaded from IEDB, we removed all duplicate peptides from the testing dataset. The alignment-ready HLA sequences were obtained from IMGT/HLA database^21^. We trained our model on 9-length peptides binding to HLA-A, HLA-B and HLA-C alleles with available HLA sequences. Totally, the training dataset contains 121,787 peptide-HLA binding peptides covering 42 HLA-A alleles (72618 samples), 49 HLA-B alleles (46915 samples) and 10 HLA-C alleles (2254 samples). The detailed information of the training and testing data are listed in Supplementary File. In this work, we only trained and tested on 9-length peptides. We plan to extend the model training on variable length peptides in future work.

### Sequence Encoding

**Figure 3:**
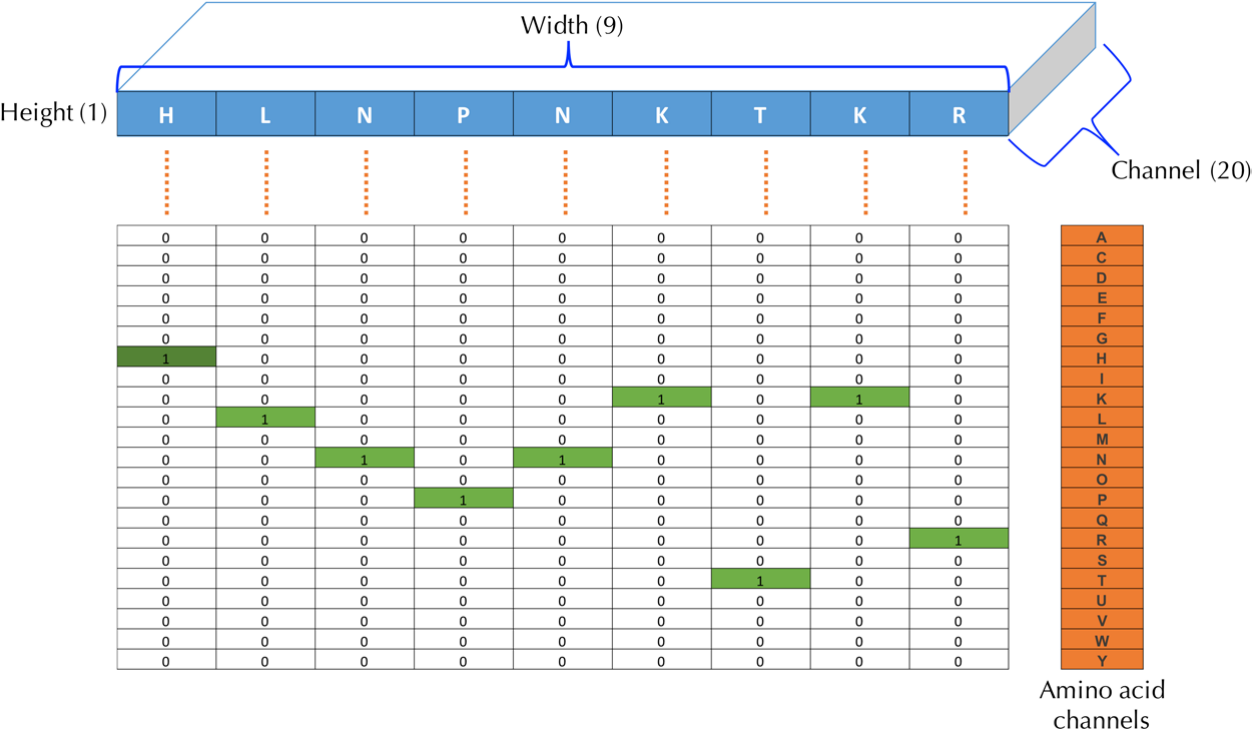
Peptide encoding example. Sequence HLNPNKTKR is encoded into a 2D tensor with dimension 1 (height) × 9 (width) × 20 (channel). Each of 20 channels represents one amino acid type and we set a channel value to 1 if the corresponding amino acid appears at this location of the input sequence.

In our DeepSeqPan model, each input is an HLA-peptide pair. For both the peptide and the HLA in an input, we use the naive one-hot encoding according to amino acids’ locations in sequences. A 9-length peptide sequence is encoded into a 2D tensor with dimension 1 × 9 × 20 where the last dimension is the number of channels and each channel represents one of 20 amino acids. Figure 3 illustrates the encoded peptide HLNPNKTKR as a 2D tensor with dimension 1 × 9 × 20. Since HLA sequences downloaded from IMGT/HLA database have variable lengths, we chose the maximum length 372 as the fixed dimension. Then we encode each aligned HLA sequence into a 2D tensor with dimension 1 × 372 × 21. The extra channel represents gaps in HLA sequences shorter than 372.

### The deep neural network model of DeepSeqPan

#### Architecture

As shown in Fig. 2, the DeepSeqPan network consists of three parts:

i. Peptide and HLA encoders. The peptide and HLA encoders convert a pair of one-hot encoded peptide and HLA sequences into two tensors with a unified dimension 1 × 9 × 10. The output tensors of two encoders are concatenated along the channel axis to generate an encoded feature tensor with dimension 1 × 9 × 20. Then this concatenated tensor will be fed into the binding context extractor in (ii). The purpose of these two encoders is extracting high-level features from raw sequences and encoding them into a feature tensor. Different from the 34 pseudo amino acid sequence encoding approach in^6^, the features and information stored inside this feature tensor are learned by the deep neural network automatically with its end-to-end training framework. The encoder of the peptide consists of two blocks of convolutional, batch normalization and LeakyReLu layers. As for the HLA encoder whose input sequence is much longer than the peptide, we used a network configuration similar to the VGG network^22^.
ii. Binding context extractor. The extractor takes into the encoded feature tensor from (i) and outputs a 2560-dimension vector. This vector is actually the binding context between a peptide and a HLA. This binding context extractor will be optimized automatically in the training stage through the backpropagation algorithm and the extraction of the binding context is done by the network itself without human involvement. Especially, in this extractor we use Locally Connected layers (as illustrated *LCBlock* in Fig. 2) instead of standard convolutional layers with weights sharing. The reason is that the encoded high-level features in the feature tensor is position related, i.e. in the encoded feature tensor with length 9, an extracted feature ${\mathscr{A}}$ located at position 1 should have different effect as it appears at position 7. Locally connected layers have the capability to capture features at specific locations since its filters at different locations do not share weights, which has been proved to be powerful in DeepFace^23^.
iii. Affinity and binding predictors. Another novel design of DeepSeqPan is that at the output layer, both the binding probabilities and the *IC*_50_ value are used as output in this stage. This is different from all other DCNN based MHC binding prediction algorithms^12,17,18^ which outputs either the binding probabilities or *IC*_50_ values. This design is not a captain’s call. Actually, at first when we were training the DeepSeqPan that only predicts *IC*_50_ values, we found it was very hard to train the network with very slow convergence. So, we added the binding probability predictor as an additional source of supervision signal with the expectation that the backpropagation algorithm can train the network easier by taking advantage of two types of losses: the classification loss and regression loss. Note that we can calculate the binary binding probability for the training samples from their *IC*_50_ binding affinity values via Equation (4). In our experiments, we used a single threshold value of 500 nM to decide whether the HLA binds to the peptide or not following the benchmark practice. However, we recognized that that the thresholds vary between different HLA molecules^6,24^ and single threshold approach is just a widely used simplification. The underlying relationship between regression outputs and classification outputs is built up naturally. In the training stage, the network needs to learn this underlying relationship in order to reduce the total loss. In that case, we argue that the classification predictor plays as a regularizer by forcing the network to predict a more accurate *IC*_50_ values.

We went through a grid search over hyperparameters setting and the detailed configurations of the layers/blocks and associated hyperparameters are described in the Supplementary File. The grid searching was performed based on the loss performance on the validation dataset.

#### Loss Function

The overall loss ${\mathcal L}$ is the sum of the regression loss ${ {\mathcal L} }_{R}$ and the classification loss ${ {\mathcal L} }_{C}$ as illustrated in Equation (1).

1 $${\boldsymbol{ {\mathcal L} }}={{\boldsymbol{ {\mathcal L} }}}_{{\boldsymbol{R}}}+{{\boldsymbol{ {\mathcal L} }}}_{{\boldsymbol{c}}}$$

For *IC*_50_ predictor, we use mean squared error (MSE, Equation (2)) as the loss function and for the binary binding predictor, the binary cross entropy loss (Equation (3)) is used.

2 $${ {\mathcal L} }_{R}=\frac{1}{N}\sum \Vert {Y}_{I{C}_{50}}-{Y^{\prime} }_{I{C}_{50}}{\Vert }^{2}$$

3 $${ {\mathcal L} }_{c}=-\,P\cdot log(P^{\prime} )-(1-P)\cdot \,\mathrm{log}(1-P^{\prime} )$$

To get binary binding labels, we use standard 500 *nM* threshold to convert a *IC*_50_ value label into a binding label:

4 $$P=\{\begin{array}{c}1,\,I{C}_{50}\le 500\\ 0,\,otherwise\end{array}$$

#### Training

We randomly split all training samples into a training set and a validation set following 4:1 ratio. Stochastic gradient descent (SGD) is employed as the optimization algorithm enabled with momentum and learning rate decay. The initial learning rate is 0.001 and the momentum factor is 0.8. It is scheduled to halve the learning rate if validation loss hasn’t improved within 5 epochs. The minimum learning rate is set to 0.00001. The training process stops if the validation loss has not improved within 15 epochs. We used Keras^25^ deep learning framework to implement our DeepSeqPan algorithm.

### Metrics and label preprocessing

Area under the curve (AUC) and Spearman’s rank correlation coefficient (SRCC) are used as evaluation metrics to compare with the public benchmark results at IEDB website^20^.

n pan-specific binding prediction modeling, the *IC*_50_ values of the peptides span a large range [0,80000]. To avoid gradient explosion issue in neural network training, we convert *IC*_50_ to *logIC*_50_ via Equation (5). The *logIC*_50_ are then used as labels during training. During inference stage, we convert the prediction results back to *IC*_50_values.

5 $$logI{C}_{50}=lo{g}_{e}I{C}_{50}$$

## Results and Discussion

### Cross-validation on the training dataset

Standard five-fold cross-validation experiments were applied to the different alleles and their combinations in the training dataset. The performance is then measured in area under a curve (AUC) and Spearman’s rank correlation coefficient (SRCC). Since our network outputs both *IC*_50_ affinity values and binding probabilities, we evaluated the performances on both outputs separately, in terms of classification performance and regression performance.

In Table 1, *BD2013* row shows the 5-fold cross-validation results of our algorithm on the original training dataset. When the calculated the metrics on all samples, DeepSeqPan achieved a high AUC of 0.94 for regression on (*IC*_50_) and an AUC of 0.94 for binary binding classification. The corresponding SRCC is 0.73 (*IC*_50_) and 0.70 (binary binding) respectively. When evaluated over the samples of HLA-A, -B and -C alleles separately, all the AUC scores are above 0.90 and SRCC scores are all above 0.60. More comprehensive allele-specific results are reported in Table S2. Besides performing cross validation on the original training dataset, we also did a cross validation on a CD-HIT^26^ filtered training dataset. We first use CD-HIT to group all peptides in our training dataset with sequence identity threshold 0.7. After this step, 20,148 unique peptide sequences are grouped into 14,812 clusters. Then for each cluster, we only keep peptide with the greatest number of samples. In this way, we got a new training dataset for cross-validation. Then we did the normal 5-fold cross-validation on this training set. In Table 1, we listed the performance of model trained on this filtered dataset in *CD-HIT BD2013* row. As shown in the table, the performance on CD-HIT filtered dataset is similar as that obtained from all training: all AUC scores are same and SRCC scores are slightly worse. Full list of allele-specific metric results is shown in Table S3 and from this table we can also find that the performance on each allele are pretty close to that of previous models trained on all samples.

**Table 1:** Five-fold cross validation on all training data and CD-HIT filtered training data.

Training Dataset	Alleles	Seq Count	*IC*_50_		Binary Binding	
			AUC	SRCC	AUC	SRCC
BD2013	All alleles	121,787	0.94	0.73	0.94	0.70
	HLA-A	72,618	0.94	0.75	0.94	0.73
	HLA-B	46,915	0.94	0.68	0.94	0.64
	HLA-C	2,254	0.89	0.70	0.89	0.69
CD-HIT BD2013	All alleles	104,449	0.94	0.71	0.94	0.68
	HLA-A	60,987	0.94	0.73	0.94	0.71
	HLA-B	41,360	0.94	0.66	0.94	0.62
	HLA-C	2,102	0.89	0.69	0.89	0.68
BD2009	All alleles	88,742	0.93	0.69	0.93	0.68
	HLA-A	57,173	0.93	0.72	0.93	0.71
	HLA-B	31,569	0.93	0.62	0.93	0.60

Furthermore, IEDB offers a dataset BD2009_cv_sr which has already been filtered and split considering data redundancy between folds. We also performed a 5-fold cross validation experiments on this dataset. In Table 1, *BD2009* row shows the results and the full list of allele-specific results are reported in Table S4. From the two tables, model shows consistent performance on BD2013 and BD2009_cv_sr.

### Evaluation on benchmark dataset

To evaluate how our DeepSeqPan performs compared to other HLA-peptide binding prediction algorithms, we applied it to the public IEDB weekly benchmark dataset upon which a set of top algorithms have been evaluated with published results.

We trained a single DeepSeqPan model on all 9-length peptides in the training dataset that bind to HLA-A, -B and -C alleles. Then this trained model was evaluated on all available IEDB weekly benchmark dataset^20^. As we inform before, the IEDB benchmark dataset has been filtered by removing duplicate samples. We compared the performance of DeepSeqPan with those of pan-specific models: NetMHCPan (2.8)^8^, NetMHCPan (3.0)^6^ and PickPocket^11^, the performances of allele-specific models: SMM^7^, NetMHC (3.4)^1^, NetMHC (4.0)^27^, ARB^20^, MHCflurry^28^ and AMMPMBEC^20^, and those of ensemble models (results are based on several different models): IEDB Consensus^20^ and NetMHCcons^29^. Metrics of compared models are listed in Table S1 which can be found in Supplementary File.

Table S1 summarized the performance of different algorithms on 64 testing datasets from IEDB benchmark database. For each dataset, we highlighted the highest AUC scores in yellow and highest SRCC scores in pink and then counted the number of datasets upon which each algorithm achieved the highest scores and put them at the last row of the table. We found that DeepSeqPan achieved the highest AUC scores in 19 records out of total 64 testing records. In 45 records that DeepSeqPan didn’t achieve the highest AUC scores, there are 28 records on which the AUC scores of DeepSeqPan are very close to the highest AUC scores within a small margin around 0.1. In terms of SRCC, DeepSeqPan obtained the highest scores on 13 records.

From Table S1, it can be found that different pan-specific and allele-specific methods have the best performance on datasets of various alleles, which implies the good performance of the ensemble methods such as NetMHCcons since they make prediction via combining results from multiple methods^29^. Our proposed DeepSeqPan could thus be a complementary tool for existing pan-specific models and it is promising to include it into the state-of-the-art ensemble prediction models to improve their performance.

### Comparison with other DCNN models

To the best of our knowledge, Kim *et al*.’s work^12^ is the only pan-specific model that employs DCNN architecture beside our proposed DeepSeqPan. It uses NetMHCPan’s pseudo sequence encoding for binding context modeling, in which a pair of peptide-HLA binding sample is encoded into a 9 (height) × 34 (width) × 18 (channel) 2D tensor. Each “pixel” in this 2D tensor represents a contacting pair of a peptide reside and a HLA residue. For two contacting residues, 9 physicochemical properties are used for each one and in total 18 values are encoded in channels. Their network structure is VGG-like and consists of 26 layers. They trained their model with binding samples on HLA-A and HLA-B alleles and it used the same dataset BD2013 as we did. To compare the performance of our DeepSeqPan with Kim’s method, we evaluated the benchmark dataset with their online server (http://jumong.kaist.ac.kr:8080/convmhc) on all its supporting alleles (HLA-A and HLA-B alleles). In total, we evaluated 54 benchmark datasets on Kim’s server and compared with ours obtained in previous benchmark evaluation and the binary prediction outputs were used to compare. Since Kim’s model was trained as a classifier, we calculated AUC scores for each testing dataset and in Table 2 we showed the average AUC scores measured based on all HLA-A or HLA-B testing dataset respectively (Detailed performance on each dataset is listed in Supplementary Files). Out of all 54 benchmark datasets, Kim’s model and our model both got an average AUC of 0.76. For HLA-A datasets, two model also obtained same average AUC of 0.74. Our model slightly out performed Kim’s model on HLA-B alleles with an average AUC of 0.80. Overall, two models achieved similar performance and in terms of performance on each allele as shown in Supplementary Files, two models obtained better performance on different sets of HLA alleles and none can dominate the other model.

**Table 2:** Comparison of Kim’s model and DeepSeqPan.

HLA	Count	AUC	
		Kim	DeepSeqPan
All	19,240	0.76	0.74
HLA-A	3,416	0.74	0.71
HLA-B	15,824	0.79	0.79

### Generalization of DeepSeqPan to binding predictions of new HLA alleles

One major advantage of pan-specific models over allele-specific models for HLA-peptide binding prediction is that it can make predictions on HLA alleles that are not included in the training dataset. This is especially useful for HLA alleles without any samples with known binding affinity values. In order to evaluate this extrapolation capability of DeepSeqPan, we setup a leave-one-allele-out (LOAO) cross validation experiment on BD2013 dataset. In each fold, we hold one allele’s samples as testing set and the rest alleles’ samples as training set. In this way, we were traying to mimic the situation where model meet unseen allele samples.

Totally, we performed a cross validation on 102 alleles. The AUC and SRCC scores of each allele are listed in Table S6. We compared this LOAO cross validation performance with that of previous random 5-fold cross validation and results are shown in Table 3. Among 102 alleles, 74 of them obtained AUC scores > 0.7on both regression and binary predictions for LOAO cross validation. While for random 5-fold cross validation, 80 alleles had AUC scores > 0.7 on regression predictions and 78 of them did on binary predictions. 50 alleles obtained AUC scores > 0.8 on regression predictions and that number is 52 on binary predictions on LOAO results. These two numbers are 72 and 70 on random 5-fold cross validation results. Regarding SRCC scores, 28 alleles obtained scores > 0.6 and 14 alleles obtained scores > 0.7 on regression predictions for LOAO cross validation. The corresponding numbers of alleles are 53 and 49 on random 5-fold cross validation results. Based on these analyses, we argue that our trained model shows reasonable generalization capability on this binding prediction problem.

**Table 3:** Performance comparison between LOAO cross validation and random 5-fold cross validation.

Metrics	Threshold	IC_50_		Binary	
		LOAO	Random 5-fold	LOAO	Random 5-fold
AUC	>0.7	74	80	74	78
	>0.8	50	72	52	70
SRCC	>0.6	28	53	26	49
	>0.7	14	34	15	32

### The binding context vector: consistency and capability

One of key design features of our DeepSeqPan model (Fig. 2) is the binding context vector aiming to capture high-level features that determine whether a peptide and a HLA bind or not and if so, how strong the binding is. Another key feature of our model is the dual outputs of the model: the binding affinity output and the binary binding probability output.

Since the binding context vector is used as input for both predicted outputs, it should be consistent for both the *IC*_50_ predictor and the binary binding predictor in^3^: for the same binding context vector, both predictors should give consistent outputs. In other words, higher binding probability should correspond to higher binding affinity values. To verify this consistency, we inspected *IC*_50_ and binary prediction outputs of all samples from previous cross-validation and benchmark evaluation experiments. The analysis results are shown in Table 4. First row of the table lists the numbers of evaluated samples in the cross-validation and benchmark evaluation experiments with 121,787 in cross-validation and 19,741 in benchmark evaluation. The second row shows the number of consistent outputs. Given a sample pair of peptide and HLA, we marked its predicted *IC*_50_ value and the predicted binding probability as consistent if both values indicate binding or not binding. An *IC*_50_value of $ < $500 *nM* or a binding probability of 0.5 or greater indicates the binding state. From the table we can observe that high consistency exists between the regression and classification outputs. For cross-validation experiments, the percentage of consistent outputs is 95.81% and for benchmark evaluation experiments, this percentage is 86.14%.

**Table 4:** Consistency inspection results.

	Cross Validation	Benchmark Evaluation
Total samples	121,787	19,741
Consistent pred.	116,688 (95.81%)	17,004 (86.14%)
Correct IC_50_ pred.	108,064 (88.73%)	11,690 (59.21%)
Correct Binary pred.	107,239 (88.05%)	10,487 (53.12%)

In last two rows of Table 4, we reported the number of correct predictions measured with the *IC*_50_ predictions and the binary prediction respectively. A predicted *IC*_50_. value or a predicted binary binding probability value is marked as a correct prediction if its real label and the prediction value indicate the same binding state: binding or not binding. Given a sample, it will be marked as binding if its IC50 value is less than the threshold (500 nM). If the sample binding affinity is labelled with t1/2 type, measured minutes less than 120 indicates the binding state For binary binding labels, a binary label of 1 means it is binding while a value of 0 means no binding. For the predicted binary binding probability, a probability value of >0.5 means binding. From Table 4, we found that both affinity and binary binding outputs obtained accuracies greater than 88% in cross-validation experiments. In benchmark experiments, the accuracy rate is 59% for *IC*_50_ predictions and binary predictions have an accuracy of 53%. The results showed that the consistency between *IC*_50_ predictions and binary predictions is high, which means that the binding context vector extracted by DeepSeqPan contains common effective features for determining binding states.

**Figure 4:**
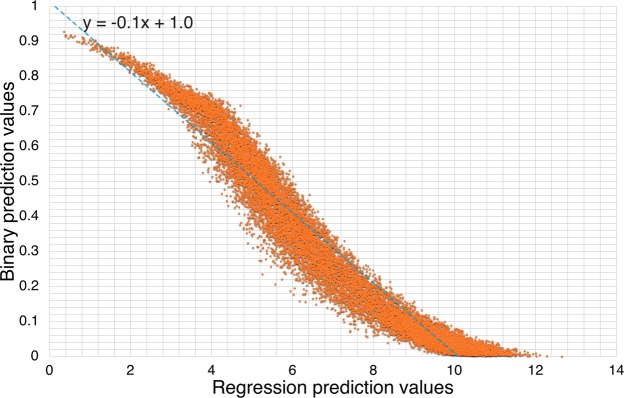
Correlation analysis between binary prediction values and regression prediction values on benchmark dataset.

In Fig. 4 we plotted the correlation between binary values and regression values predicted on benchmark dataset. Each dot in this plot represents a testing sample’s two prediction values by DeepSeqPan. The *x* is value is the predicted logIC50 and the *y* axis value is the predicted binary binding probability. The Pearson correlation value calculated is −0.97. We also fitted a linear function into these correlation data and the fitted linear function is *y* = −0.1*x* + 0.1. From the figure, it shows that two output values have very strong correlation when both indicate very strong binding or very weak binding (upper left part and lower right part). It displays weak correlation when the two predicted values indicate the binding is neither strong or weak.

Though the results show that the two output values have a pretty strong correlation overall. There are some cases they will give contradicting predictions. We think the major reason behind this is that in the training dataset, there’re some *edge* samples. Following the 500 nM hard convention value, for samples whose IC_50_ values are slightly above 500 (e.g. 502.03), their binary labels will be 0. And for those samples whose IC_50_ values are slightly below 500 (e.g. 498.33), their binary labels will be 1. After training, when the model does prediction on those similar edge samples in testing dataset, the predicted regression values and binary labels are easy to contradict to each other. It can be seen that around hard convention line (*x* = 6.20) in Fig. 3, the correlation is almost weakest. In practical usage, users should be careful on predicted regression values around 500 and measure the result based on two outputs. But if users only care strong binding samples, according to our correlation analysis on benchmark testing, the two values show strong correlation in strong binding cases. That will not be a problem.

Tables 1, 4 and S1 together showed that the binding context vector learned via the end-to-end learning framework of the DCNN is predictor-independent and has captured information related to HLA-peptide binding. Its effectiveness in HLA binding prediction may be explained by its capability to capture position related information such as when there’s an amino acid A in HLA at position 37 and at the same time there’s an amino acid L in peptide at position 5, the binding affinity is high. This binding feature extraction is similar to DCNN architectures like VGG16^22^, ResNet^30^ of computer vision in which an input image will be represented as a high-dimension vector (4096 in VGG16 and 1000 in ResNet) in the final stage of the neural network. Due to time and hardware resource constraints, our current version of DeepSeqPan only uses one-hot encoding information. No other information such as physical properties of amino acids are encoded into input tensors, which however can further improve the performance of DeepSeqPan if properly encoded by capturing more relevant and rich binding contexts.

## Conclusion

In this work, we proposed, DeepSeqPan, a novel deep convolutional neural network model for pan-specific HLA-peptide binding affinity prediction. This model is characterized by its capability of binding prediction with only the raw amino acid sequences of the peptide and the HLA, which makes it applicable to HLA-peptide binding prediction for HLA alleles without structural information. This is achieved by a novel sequence-based encoding of the peptide-HLA binding context, a binding context feature extractor, and the dual outputs with both binding affinity and binding probability predictions. Extensive evaluation of DeepSeqPan on public benchmark experiments showed that our model achieves state-of-the-art performance on a variety of HLA allele datasets.

Our model contributes to the study of MHC-peptide binding prediction in a few special ways. First, our experiments showed that it is possible to extrapolate the binding prediction capability to unseen HLA alleles, which is important for pan-specific models. Second, our sequence-only based binding context encoding is complementary to the pseudo sequence encoding, which is currently the only encoding method used in pan-specific models for class I MHC-peptide binding affinity prediction. This has the potential to further improve the state-of-the-art prediction models such as the pan-specific model NetMHCSpan. It showed the importance of sufficient amount of training data to achieve high prediction performance for deep learning models.

Our current work can be further improved in a number of ways. First, in this work, only one-hot encoding is used for representing the input peptide and HLA protein. However, this can be improved by properly encoding more features such as physicochemical properties of amino acids into input tensors. Moreover, our proposed sequence-based DCNN architecture for protein-peptide binding is universal and can be adapted to other similar binding problems such as protein-DNA, protein-RNA and protein-ligand/drug bindings.

## Supplementary information

Supplementary Files

## Data Availability

The implementation code and trained models are freely available at https://github.com/pcpLiu/DeepSeqPan.
