# Supplementary material for: DeepSeqPan, a novel deep convolutional neural network model for pan-specific class I HLA-peptide binding affinity prediction
Source: Sci Rep. 2019 Jan 28;9:794. doi: 10.1038/s41598-018-37214-1 (PMC6349913; doi:10.1038/s41598-018-37214-1)
Supplement: Supplementary file 1 — Supplementary Files [file 41598_2018_37214_MOESM1_ESM.pdf]

Supplementary Files of

DeepSeqPan, a novel deep convolutional neural network model for pan-specific class I HLA-peptide binding affinity prediction

Zhonghao Liu[1], Yuxin Cui[1], Zheng Xiong[1], Alierza Nasiri[1], Ansi Zhang[2], Jianjun Hu[1,2*]

1 Department of Computer Science and Engineering, University of South Carolina, 29201 Columbia, SC, United States
2 School of Mechanical Engineering, Guizhou University, 50033 Guiyang, Guizhou, China

***Corresponding, jianjunh@cse.sc.edu**

1 Availability

All dataset, source code and trained models are included in our Github repository https://github.com/pcpLiu/DeepSeqPan.

2 Dataset

2.1 Training dataset

The training dataset is downloaded from http://tools.iedb.org/main/datasets/, binding_data_2013.zip.

2.2 Benchmark testing dataset

We downloaded all available weekly benchmark dataset from http://tools.iedb.org/auto_bench/mhci/weekly/ including from 2014-03-21 to 2018-01-26. Since there are quite a lot duplicates between training and benchmark dataset, we removed all duplicates in benchmark dataset. The filtered dataset can be found in Github repository.

3 Benchmark testing result

Table S1, we listed all method's evalutions results on benchmark dataset.

4 5-fold Cross validation results on BD2013 and BD2009

The 5-fold cross validation results measured for each allele on BD2013 are listed in the Table S2. In the table, some AUC scores are N/A because of that in training dataset, there are few alleles coming with 0 positive samples. So it is not applicable to calculate AUC scores. For alleles HLA-B*27:10 and HLA-A*02:04, the SRCC cannot be calculated since their number of samples ≤ 2 which is not a calculable situation for spearmanr function defined in Scipy (https://docs.scipy.org/doc/scipy-0.18.1/reference/generated/scipy.stats.mstats.spearmanr.html). For alleles

Table S1. Evaluation on benchmark database. Red/Yellow highlighted number(s) are best AUC/SRCC scores in that record. Sorted by IEDB Ref ID.

Measure					Pan-specific								Allele-specific												Ensemble			
HLA	IEDB Ref	type	Positive	Negative	DeepSeqPan		NetMHCpan 2.8		NetMHCpan 3.0		PickPocket		SMM		NetMHC 3.4		NetMHC 4.0		ARB		SMMPMBEC		MHCflurry		IEDB Consensus		NetMHCcons	
					AUC	SRCC	AUC	SRCC	AUC	SRCC	AUC	SRCC	AUC	SRCC	AUC	SRCC	AUC	SRCC	AUC	SRCC	AUC	SRCC	AUC	SRCC	AUC	SRCC	AUC	SRCC
HLA-B*27-03	315174	Binary	6	4	0.58	0.14	0.92	0.71	-	-	0.88	0.64	-	-	-	-	-	-	-	-	-	-	-	-	-	-	0.92	0.71
HLA-A*66-01	315312	Binary	14	2	0.11	-0.45	-	-	0.54	0.04	0.68	0.20	0.68	0.20	-	-	0.25	-0.29	-	-	1.00	0.57	0.86	0.41	0.64	0.16	0.61	0.12
HLA-A*31-01	315312	Binary	4	4	0.88	0.65	-	-	0.88	0.65	0.88	0.65	0.88	0.65	-	-	0.88	0.65	-	-	0.88	0.65	0.88	0.65	0.88	0.65	0.88	0.65
HLA-B*07-02	1026371	t1/2	10	23	0.93	0.66	0.93	0.81	-	-	-	-	0.93	0.72	0.93	0.77	-	-	0.72	0.44	-	-	-	-	-	-	-	-
HLA-A*02-01	1026371	t1/2	16	18	0.75	0.41	0.75	0.42	-	-	-	-	0.77	0.45	0.76	0.45	-	-	0.75	0.45	-	-	-	-	-	-	-	-
HLA-B*07-02	1026840	Binary	20	268	0.83	0.29	0.87	0.32	-	-	-	-	0.88	0.33	0.88	0.33	-	-	0.85	0.31	-	-	-	-	-	-	-	-
HLA-C*07-01	1026840	Binary	31	408	0.67	0.15	0.78	0.25	-	-	-	-	0.65	0.13	0.76	0.23	-	-	-	-	-	-	-	-	-	-	-	-
HLA-C*07-01	1026840	IC$_{50}$	12	6	0.32	-0.29	0.54	-0.18	-	-	-	-	0.39	-0.01	0.61	0.17	-	-	-	-	-	-	-	-	-	-	-	-
HLA-A*30-01	1026840	Binary	8	339	0.83	0.17	0.81	0.16	-	-	-	-	0.79	0.15	0.77	0.14	-	-	0.71	0.11	-	-	-	-	-	-	-	-
HLA-B*58-01	1026840	Binary	45	388	0.84	0.36	0.86	0.38	-	-	-	-	0.87	0.40	0.85	0.37	-	-	0.84	0.36	-	-	-	-	-	-	-	-
HLA-B*58-01	1026840	IC$_{50}$	17	17	0.52	0.19	0.72	0.38	-	-	-	-	0.70	0.38	0.68	0.33	-	-	0.57	0.26	-	-	-	-	-	-	-	-
HLA-B*58-01	1026840	t1/2	4	30	0.56	0.03	0.54	0.15	-	-	-	-	0.53	0.08	0.54	0.16	-	-	0.50	0.12	-	-	-	-	-	-	-	-
HLA-A*30-02	1026840	Binary	109	251	0.72	0.35	0.77	0.42	-	-	-	-	0.73	0.36	0.75	0.40	-	-	0.65	0.25	-	-	-	-	-	-	-	-
HLA-A*30-02	1026840	IC$_{50}$	36	20	0.64	0.21	0.47	0.01	-	-	-	-	0.54	0.12	0.59	0.13	-	-	0.64	0.27	-	-	-	-	-	-	-	-
HLA-A*30-02	1026840	t1/2	14	42	0.43	0.15	0.50	0.05	-	-	-	-	0.50	0.07	0.55	0.19	-	-	0.52	0.15	-	-	-	-	-	-	-	-
HLA-A*68-01	1026840	Binary	43	393	0.82	0.33	0.87	0.38	-	-	-	-	0.86	0.37	0.87	0.38	-	-	0.78	0.34	-	-	-	-	-	-	-	-
HLA-A*68-01	1026840	IC$_{50}$	13	22	0.76	0.44	0.84	0.63	-	-	-	-	0.79	0.62	0.84	0.65	-	-	0.77	0.53	-	-	-	-	-	-	-	-
HLA-A*68-01	1026840	t1/2	19	16	0.40	-0.13	0.32	-0.32	-	-	-	-	0.25	-0.42	0.27	-0.41	-	-	0.31	-0.39	-	-	-	-	-	-	-	-
HLA-A*24-02	1026840	Binary	40	306	0.81	0.34	0.85	0.39	-	-	-	-	0.82	0.36	0.85	0.38	-	-	0.83	0.37	-	-	-	-	-	-	-	-
HLA-A*24-02	1026840	IC$_{50}$	12	7	0.54	0.16	0.63	0.18	-	-	-	-	0.76	0.39	0.61	0.19	-	-	0.45	0.03	-	-	-	-	-	-	-	-
HLA-A*02-01	1026840	Binary	63	278	0.82	0.42	0.89	0.52	-	-	-	-	0.88	0.51	0.87	0.49	-	-	0.88	0.51	-	-	-	-	-	-	-	-
HLA-A*02-01	1026840	IC$_{50}$	13	9	0.69	0.31	0.72	0.36	-	-	-	-	0.68	0.37	0.63	0.29	-	-	0.74	0.45	-	-	-	-	-	-	-	-
HLA-A*02-01	1026840	t1/2	6	16	0.74	0.33	0.73	0.43	-	-	-	-	0.74	0.37	0.67	0.29	-	-	0.69	0.46	-	-	-	-	-	-	-	-
HLA-C*07-02	1026891	Binary	7	13	0.60	0.17	0.65	0.25	-	-	-	-	0.74	0.39	0.75	0.41	-	-	-	-	-	-	-	-	-	-	-	-
HLA-A*11-01	1026891	Binary	14	2	0.21	-0.33	0.43	-0.08	-	-	-	-	0.50	0.00	0.46	-0.04	-	-	0.14	-0.41	-	-	-	-	-	-	-	-
HLA-B*58-01	1026891	Binary	12	8	0.59	0.16	0.88	0.64	-	-	-	-	0.76	0.44	0.88	0.64	-	-	0.84	0.58	-	-	-	-	-	-	-	-
HLA-B*40-01	1026891	Binary	9	10	0.81	0.54	0.92	0.73	-	-	-	-	0.86	0.62	0.91	0.71	-	-	0.87	0.64	-	-	-	-	-	-	-	-
HLA-A*24-02	1026891	Binary	15	4	0.50	0.00	0.63	0.19	-	-	-	-	0.57	0.09	0.53	0.05	-	-	0.55	0.07	-	-	-	-	-	-	-	-
HLA-B*58-01	1026897	Binary	3	19	0.77	0.32	0.74	0.28	-	-	-	-	0.81	0.37	0.75	0.30	-	-	0.67	0.20	-	-	-	-	-	-	-	-
HLA-B*40-01	1026897	Binary	4	11	0.82	0.49	0.75	0.38	-	-	-	-	0.84	0.52	0.75	0.38	-	-	0.75	0.38	-	-	-	-	-	-	-	-
HLA-A*02-01	1027079	Binary	8	7	0.77	0.46	0.77	0.46	-	-	-	-	0.68	0.31	0.75	0.43	-	-	0.68	0.31	-	-	-	-	-	-	-	-
HLA-B*15-02	1027131	Binary	11	3	1.00	0.71	1.00	0.71	-	-	1.00	0.71	0.91	0.58	1.00	0.71	-	-	1.00	0.72	1.00	0.71	1.00	0.71	1.00	0.71	1.00	0.71
HLA-A*02-01	1027471	Binary	5	38	0.94	0.49	0.84	0.38	-	-	-	-	0.83	0.36	0.81	0.34	-	-	0.81	0.35	-	-	-	-	-	-	-	-
HLA-A*02-01	1027588	Binary	7	11	0.87	0.63	0.83	0.56	-	-	-	-	0.84	0.58	0.82	0.55	-	-	0.75	0.43	-	-	-	-	-	-	-	-
HLA-C*03-03	1028228	IC$_{50}$	5	5	1.00	0.77	0.68	0.68	-	-	0.64	0.47	0.68	0.46	0.68	0.46	-	-	-	-	0.76	0.67	-	-	0.68	0.46	0.68	0.56
HLA-A*01-01	1028282	t1/2	3	3	1.00	0.84	1.00	0.90	-	-	1.00	0.90	1.00	0.81	1.00	0.90	-	-	1.00	0.75	1.00	0.90	-	-	1.00	0.79	1.00	0.90
HLA-A*02-01	1028285	t1/2	107	28	0.78	0.49	0.76	0.52	-	-	0.82	0.59	0.79	0.56	0.74	0.51	-	-	0.79	0.49	0.78	0.55	-	-	0.78	0.56	0.76	0.53
HLA-A*11-01	1028287	t1/2	141	78	0.80	0.58	0.90	0.76	-	-	0.80	0.51	0.90	0.75	0.90	0.74	-	-	0.87	0.67	0.90	0.75	-	-	0.91	0.76	0.91	0.76
HLA-A*03-01	1028288	t1/2	53	168	0.81	0.50	0.85	0.58	-	-	0.77	0.48	0.86	0.56	0.85	0.57	-	-	0.85	0.54	0.86	0.57	-	-	0.86	0.58	0.86	0.59
HLA-A*24-02	1028289	t1/2	146	277	0.74	0.48	0.79	0.56	-	-	0.81	0.60	0.80	0.57	0.78	0.57	-	-	0.76	0.50	0.80	0.58	-	-	0.80	0.59	0.80	0.60
HLA-A*26-01	1028290	t1/2	0	3	-	-0.5	-	-0.5	-	-	-	0.50	-	0.50	-	-0.5	-	-	-	0.50	-	0.50	-	-	-	0.50	-	-0.50
HLA-B*07-02	1028291	t1/2	60	76	0.83	0.67	0.89	0.78	-	-	0.81	0.69	0.88	0.76	0.87	0.76	-	-	0.80	0.63	0.88	0.76	-	-	0.88	0.76	0.89	0.78
HLA-B*35-01	1028292	t1/2	135	228	0.79	0.52	0.83	0.62	-	-	0.83	0.60	0.81	0.58	0.83	0.62	-	-	0.74	0.45	0.81	0.58	-	-	0.82	0.59	0.84	0.64
HLA-B*15-01	1028293	t1/2	433	137	0.59	0.19	0.74	0.43	-	-	0.61	0.22	0.71	0.40	-	-	-	-	0.59	0.20	0.71	0.40	-	-	0.70	0.38	0.74	0.43
HLA-B*40-01	1028294	t1/2	0	9	-	0.98	-	0.96	-	-	-	0.93	-	0.86	-	0.98	-	-	-	0.73	-	0.93	-	-	-	-	-	0.98
HLA-B*07-02	1028553	IC$_{50}$	3	19	0.89	0.81	0.89	0.66	-	-	0.89	0.62	0.89	0.62	0.92	0.76	-	-	0.88	0.55	0.88	0.62	-	-	0.92	0.72	0.91	0.72
HLA-A*02-01	1028553	IC$_{50}$	2	20	0.88	0.52	0.92	0.62	-	-	0.83	0.58	0.85	0.59	0.91	0.70	-	-	0.90	0.69	0.83	0.62	-	-	0.84	0.62	0.92	0.64
HLA-B*07-02	1028554	IC$_{50}$	6	46	0.71	0.61	0.77	0.62	-	-	0.74	0.70	0.85	0.66	0.88	0.70	-	-	0.76	0.65	0.86	0.70	-	-	0.88	0.70	0.86	0.73
HLA-B*57-01	1028554	IC$_{50}$	10	43	0.80	0.43	0.86	0.62	-	-	0.85	0.44	0.77	0.33	0.94	0.52	-	-	0.63	0.12	0.77	0.29	-	-	0.87	0.43	0.92	0.56
HLA-B*35-01	1028554	IC$_{50}$	3	53	0.64	0.20	0.68	0.36	-	-	0.50	0.28	0.59	0.21	0.57	0.27	-	-	0.64	0.26	0.53	0.20	-	-	0.65	0.30	0.64	0.36
HLA-B*44-03	1028554	IC$_{50}$	3	43	0.77	0.31	0.61	0.46	-	-	0.64	0.43	0.75	0.47	0.65	0.56	-	-	0.56	0.25	0.76	0.55	-	-	0.64	0.51	0.64	0.55
HLA-A*02-01	1028554	IC$_{50}$	7	37	0.75	0.44	0.89	0.70	-	-	0.88	0.51	0.89	0.58	0.82	0.62	-	-	0.76	0.51	0.86	0.55	-	-	0.84	0.58	0.89	0.69
HLA-B*07-02	1028928	Binary	10	1	1.00	0.50	1.00	0.50	-	-	1.00	0.50	1.00	0.50	1.00	0.50	-	-	1.00	0.50	1.00	0.50	-	-	1.00	0.52	1.00	0.50
HLA-A*02-01	1028928	Binary	9	2	1.00	0.67	0.94	0.60	-	-	0.92	0.56	0.94	0.60	0.94	0.60	-	-	0.94	0.60	0.94	0.60	-	-	0.94	0.60	0.94	0.60
HLA-B*57-01	1029061	IC$_{50}$	3	14	0.83	0.63	0.88	0.60	-	-	0.86	0.72	0.88	0.64	0.81	0.64	-	-	0.40	0.12	0.81	0.65	0.90	0.67	0.83	0.64	0.83	0.57
HLA-B*27-04	1029125	Binary	14	7	0.89	0.63	0.94	0.72	-	-	0.83	0.53	-	-	-	-	-	-	-	-	-	-	-	-	-	-	0.94	0.72
HLA-B*27-06	1029125	Binary	13	8	0.75	0.42	0.75	0.42	-	-	0.80	0.50	-	-	-	-	-	-	-	-	-	-	-	-	-	-	0.75	0.42
HLA-B*27-05	1029125	Binary	14	7	0.89	0.63	0.96	0.75	-	-	0.91	0.67	0.91	0.67	0.94	0.72	-	-	0.88	0.62	0.96	0.75	0.89	0.63	0.91	0.68	0.96	0.75
HLA-A*02-01	1029824	Binary	56	21	0.55	0.07	0.55	0.07	-	-	0.55	0.08	0.57	0.10	0.57	0.11	-	-	0.52	0.04	0.57	0.10	0.57	0.11	0.57	0.11	0.56	0.09
HLA-B*38-01	1029957	IC$_{50}$	0	5	-	-0.70	-	0.00	-	-	-	-0.10	-	-0.10	-	-0.10	-	-	-	-0.50	-	-0.10	-	0.30	-	-0.10	-	0.00
HLA-B*07-02	1031253	IC$_{50}$	7	6	1.00	0.93	-	-	1.00	0.97	1.00	0.87	1.00	0.96	-	-	1.00	0.98	-	-	1.00	0.97	1.00	0.91	1.00	0.97	1.00	0.97
HLA-A*03-01	1031253	IC$_{50}$	9	5	0.93	0.87	-	-	0.93	0.78	0.96	0.71	0.87	0.69	-	-	0.96	0.79	-	-	0.91	0.75	0.76	0.56	0.91	0.74	0.91	0.76
HLA-B*27-05	1031253	IC$_{50}$	7	5	0.77	0.67	-	-	0.63	0.59	0.60	0.49	0.63	0.44	-	-	0.69	0.58	-	-	0.60	0.46	0.57	0.48	0.63	0.49	0.66	0.62
HLA-B*27-05	1031959	Binary	9,364	4,176	0.60	0.16	-	-	0.60	0.16	0.60	0.16	-	-	-	-	-	-	-	-	-	-	-	-	-	-	0.60	0.16
Highlighted					19	13	23	19	3	2	10	7	16	10	16	14	3	2	4	6	8	5	5	3	10	5	16	15

HLA-A*02:10, the Spearmanr function gives nan result because all samples are labeled as same value which is also a non-calculable situation define in Scipy. Similar, the 5-fold cross validation results measured for each allele on all CD-HIT filtered BD2013 are listed in the Table S3. The 5-fold cross validation results on BD2009 (cv_sr) are listed in Table S4.

5 Leave-one-allele-out cross validation on BD2013

The leave-one-allele-out cross validation results on BD2013 are listed in Table S6.

6 Compared with Kim's DCNN model

As show in Table S7, we listed AUC scores of Kim's DCNN model and our proposed DeepSeqPan.

7 DeepSeqPan network details

7.1 Layer configuration

FC *N*. A fully connected layer with N hidden units.
Dropout. We used 0.5 as dropout rate.
ConvBlock *N*. A ConvBlock N consists of 4 layers in order:

1. A 2D Convolutional layer with N filters of size 1×3;
2. A Batch normalization layer;
3. A 2D Convolutional layer with N filters of size 1×3;
4. A Max pooling with kernel size 1×2.

LCBlock *N*. A LCBlock N consists of 3 layers in order:

1. A 2D Locally Connected layer with N filters of size 1×2;
2. A Batch normalization layer;
3. A LeakyReLU activation layer.

7.2 Parameters initialization and regularizations

For all trainable layers, we use uniform distribution in range $[-0.05, 0.05]$ to randomly generate initial values of all parameters. And we disabled bias parameters for all trainable layers. $l2$ regularizer is used for all parameter training and the control factor is 0.1.

Table S2. 5-fold cross validation measured for each allele (BD2013, randomly split)

	IC_{50}		Binary			IC_{50}		Binary	
HLA	AUC	SRCC	AUC	SRCC	HLA	AUC	SRCC	AUC	SRCC
HLA-A*01:01	0.93	0.57	0.94	0.55	HLA-B*15:09	0.88	0.47	0.89	0.47
HLA-A*02:01	0.95	0.81	0.95	0.81	HLA-B*15:17	0.93	0.69	0.94	0.69
HLA-A*02:02	0.93	0.86	0.93	0.86	HLA-B*15:42	0.82	0.04	0.65	-0.01
HLA-A*02:03	0.95	0.82	0.95	0.82	HLA-B*18:01	0.90	0.52	0.89	0.48
HLA-A*02:04	N/A	N/A	N/A	N/A	HLA-B*27:01	0.00	-1.00	0.00	-1.00
HLA-A*02:05	0.99	0.84	0.98	0.81	HLA-B*27:02	N/A	-0.50	N/A	-0.50
HLA-A*02:06	0.91	0.80	0.91	0.79	HLA-B*27:03	N/A	0.03	N/A	-0.01
HLA-A*02:07	0.76	0.75	0.79	0.79	HLA-B*27:04	N/A	1.00	N/A	1.00
HLA-A*02:10	N/A	N/A	N/A	N/A	HLA-B*27:05	0.94	0.63	0.94	0.59
HLA-A*02:11	0.96	0.80	0.96	0.80	HLA-B*27:06	N/A	-1.00	N/A	-1.00
HLA-A*02:12	0.97	0.77	0.97	0.77	HLA-B*27:10	N/A	N/A	N/A	N/A
HLA-A*02:16	0.98	0.68	0.98	0.68	HLA-B*27:20	0.56	0.52	0.54	0.56
HLA-A*02:17	0.68	0.41	0.69	0.41	HLA-B*35:01	0.90	0.72	0.91	0.71
HLA-A*02:19	0.96	0.66	0.96	0.66	HLA-B*35:03	0.87	0.50	0.90	0.54
HLA-A*02:50	1.00	0.88	0.99	0.87	HLA-B*37:01	0.48	0.01	0.61	0.13
HLA-A*03:01	0.93	0.73	0.93	0.72	HLA-B*38:01	0.98	0.81	0.98	0.80
HLA-A*03:02	0.69	0.57	0.68	0.57	HLA-B*39:01	0.93	0.62	0.93	0.61
HLA-A*03:19	0.87	0.55	0.87	0.56	HLA-B*40:01	0.97	0.63	0.97	0.59
HLA-A*11:01	0.95	0.77	0.95	0.76	HLA-B*40:02	0.90	0.76	0.90	0.75
HLA-A*11:02	1.00	0.77	1.00	0.77	HLA-B*40:13	0.64	0.48	0.61	0.46
HLA-A*23:01	0.93	0.71	0.92	0.68	HLA-B*42:01	0.94	0.77	0.94	0.77
HLA-A*24:02	0.90	0.67	0.90	0.66	HLA-B*42:02	0.84	0.64	0.82	0.63
HLA-A*24:03	0.96	0.70	0.96	0.68	HLA-B*44:02	0.95	0.60	0.94	0.55
HLA-A*25:01	0.98	0.47	0.99	0.47	HLA-B*44:03	0.94	0.82	0.95	0.82
HLA-A*26:01	0.93	0.52	0.93	0.49	HLA-B*45:01	0.92	0.67	0.91	0.66
HLA-A*26:02	0.96	0.74	0.96	0.74	HLA-B*45:06	0.81	0.14	0.71	0.17
HLA-A*26:03	0.93	0.52	0.94	0.54	HLA-B*46:01	0.92	0.44	0.93	0.44
HLA-A*29:02	0.88	0.65	0.87	0.63	HLA-B*48:01	0.92	0.49	0.92	0.50
HLA-A*30:01	0.91	0.70	0.92	0.70	HLA-B*51:01	0.92	0.58	0.92	0.55
HLA-A*30:02	0.82	0.63	0.82	0.62	HLA-B*52:01	0.58	0.29	0.50	0.18
HLA-A*31:01	0.93	0.74	0.93	0.74	HLA-B*53:01	0.92	0.78	0.93	0.77
HLA-A*32:01	0.85	0.71	0.85	0.72	HLA-B*54:01	0.90	0.73	0.90	0.73
HLA-A*32:07	0.81	0.53	0.82	0.53	HLA-B*57:01	0.96	0.62	0.96	0.59
HLA-A*32:15	0.51	0.38	0.50	0.36	HLA-B*57:02	0.84	0.66	0.80	0.62
HLA-A*33:01	0.92	0.73	0.92	0.73	HLA-B*57:03	0.97	0.74	0.97	0.75
HLA-A*66:01	0.83	0.41	0.84	0.35	HLA-B*58:01	0.95	0.69	0.96	0.68
HLA-A*68:01	0.90	0.79	0.90	0.79	HLA-B*58:02	0.55	0.51	0.59	0.55
HLA-A*68:02	0.92	0.70	0.92	0.69	HLA-B*73:01	0.64	0.36	0.66	0.37
HLA-A*68:23	0.77	0.53	0.78	0.54	HLA-B*81:01	0.92	0.76	0.93	0.76
HLA-A*69:01	0.94	0.51	0.94	0.53	HLA-B*83:01	0.93	0.53	0.93	0.53
HLA-A*74:01	0.70	0.45	0.61	0.33	HLA-C*03:03	0.80	0.59	0.79	0.58
HLA-A*80:01	0.94	0.55	0.95	0.55	HLA-C*04:01	0.52	-0.09	0.58	-0.15
HLA-B*07:02	0.95	0.72	0.95	0.72	HLA-C*05:01	0.91	0.74	0.92	0.75
HLA-B*08:01	0.91	0.66	0.91	0.65	HLA-C*06:02	0.89	0.74	0.89	0.74
HLA-B*08:02	0.96	0.42	0.96	0.44	HLA-C*07:01	0.84	0.61	0.84	0.61
HLA-B*08:03	0.92	0.35	0.95	0.38	HLA-C*07:02	0.74	0.42	0.73	0.41
HLA-B*14:01	0.74	0.43	0.74	0.44	HLA-C*08:02	0.64	0.28	0.63	0.25
HLA-B*14:02	0.84	0.51	0.85	0.43	HLA-C*12:03	0.62	0.28	0.61	0.29
HLA-B*15:01	0.90	0.67	0.90	0.67	HLA-C*14:02	0.67	0.28	0.67	0.29
HLA-B*15:02	0.77	0.53	0.77	0.53	HLA-C*15:02	0.79	0.52	0.80	0.55
HLA-B*15:03	0.90	0.75	0.90	0.75					

Table S3. 5-fold cross validation measured for each allele (CD-HIT filtered BD2013, randomly split)

	IC_{50}		Binary			IC_{50}		Binary	
Allele	AUC	SRCC	AUC	SRCC	Allele	AUC	SRCC	AUC	SRCC
HLA-A*01:01	0.94	0.57	0.94	0.55	HLA-B*15:09	0.92	0.47	0.92	0.49
HLA-A*02:01	0.95	0.81	0.95	0.80	HLA-B*15:17	0.93	0.69	0.93	0.69
HLA-A*02:02	0.93	0.85	0.93	0.85	HLA-B*15:42	0.86	0.06	0.69	0.00
HLA-A*02:03	0.95	0.80	0.95	0.79	HLA-B*18:01	0.89	0.50	0.89	0.46
HLA-A*02:04	N/A	N/A	N/A	N/A	HLA-B*27:01	N/A	B/A	N/A	N/A
HLA-A*02:05	1.00	0.85	1.00	0.81	HLA-B*27:02	N/A	0.60	N/A	0.60
HLA-A*02:06	0.91	0.79	0.90	0.77	HLA-B*27:03	N/A	-0.05	N/A	-0.01
HLA-A*02:07	0.87	0.79	0.85	0.81	HLA-B*27:04	N/A	1.00	N/A	1.00
HLA-A*02:10	N/A	N/A	N/A	N/A	HLA-B*27:05	0.94	0.60	0.94	0.57
HLA-A*02:11	0.95	0.80	0.96	0.80	HLA-B*27:06	N/A	1.00	N/A	1.00
HLA-A*02:12	0.97	0.77	0.97	0.77	HLA-B*27:10	N/A	N/A	N/A	N/A
HLA-A*02:16	0.97	0.68	0.97	0.69	HLA-B*27:20	0.69	0.50	0.68	0.47
HLA-A*02:17	0.66	0.38	0.66	0.38	HLA-B*35:01	0.90	0.69	0.90	0.68
HLA-A*02:19	0.96	0.65	0.96	0.65	HLA-B*35:03	0.81	0.50	0.83	0.54
HLA-A*02:50	0.98	0.86	0.99	0.87	HLA-B*37:01	0.73	0.37	0.66	0.25
HLA-A*03:01	0.93	0.72	0.94	0.71	HLA-B*38:01	0.97	0.59	0.97	0.59
HLA-A*03:02	0.45	0.54	0.45	0.53	HLA-B*39:01	0.93	0.62	0.94	0.62
HLA-A*03:19	0.67	0.20	0.69	0.23	HLA-B*40:01	0.96	0.59	0.97	0.55
HLA-A*11:01	0.95	0.77	0.95	0.75	HLA-B*40:02	0.89	0.73	0.87	0.69
HLA-A*11:02	0.90	0.79	0.95	0.94	HLA-B*40:13	0.63	0.56	0.60	0.56
HLA-A*23:01	0.92	0.68	0.91	0.64	HLA-B*42:01	0.94	0.83	0.96	0.83
HLA-A*24:02	0.90	0.67	0.90	0.66	HLA-B*42:02	0.72	0.43	0.72	0.41
HLA-A*24:03	0.96	0.67	0.96	0.65	HLA-B*44:02	0.94	0.56	0.94	0.51
HLA-A*25:01	0.98	0.48	0.98	0.48	HLA-B*44:03	0.94	0.79	0.94	0.79
HLA-A*26:01	0.93	0.50	0.93	0.47	HLA-B*45:01	0.91	0.65	0.91	0.63
HLA-A*26:02	0.97	0.76	0.96	0.75	HLA-B*45:06	0.71	0.09	0.52	0.08
HLA-A*26:03	0.95	0.52	0.94	0.53	HLA-B*46:01	0.90	0.41	0.92	0.42
HLA-A*29:02	0.87	0.62	0.86	0.59	HLA-B*48:01	0.96	0.54	0.95	0.54
HLA-A*30:01	0.91	0.67	0.91	0.67	HLA-B*51:01	0.92	0.54	0.93	0.51
HLA-A*30:02	0.83	0.64	0.82	0.62	HLA-B*52:01	0.67	0.36	0.50	0.04
HLA-A*31:01	0.93	0.73	0.93	0.73	HLA-B*53:01	0.90	0.70	0.90	0.70
HLA-A*32:01	0.84	0.67	0.84	0.68	HLA-B*54:01	0.89	0.70	0.89	0.69
HLA-A*32:07	0.72	0.33	0.73	0.31	HLA-B*57:01	0.96	0.59	0.96	0.56
HLA-A*32:15	0.54	0.27	0.54	0.24	HLA-B*57:02	0.92	0.84	0.83	0.82
HLA-A*33:01	0.92	0.71	0.91	0.71	HLA-B*57:03	1.00	0.46	0.97	0.40
HLA-A*66:01	0.86	0.42	0.85	0.39	HLA-B*58:01	0.95	0.64	0.95	0.63
HLA-A*68:01	0.88	0.78	0.88	0.77	HLA-B*58:02	0.72	0.43	0.74	0.47
HLA-A*68:02	0.92	0.66	0.92	0.66	HLA-B*73:01	0.71	0.51	0.70	0.48
HLA-A*68:23	0.75	0.54	0.75	0.54	HLA-B*81:01	0.94	0.81	0.89	0.78
HLA-A*69:01	0.92	0.50	0.92	0.51	HLA-B*83:01	0.93	0.55	0.93	0.55
HLA-A*74:01	0.79	0.68	0.79	0.68	HLA-C*03:03	0.84	0.60	0.82	0.58
HLA-A*80:01	0.94	0.57	0.94	0.57	HLA-C*04:01	0.47	-0.06	0.42	-0.10
HLA-B*07:02	0.95	0.71	0.95	0.71	HLA-C*05:01	0.92	0.75	0.92	0.74
HLA-B*08:01	0.91	0.66	0.91	0.65	HLA-C*06:02	0.87	0.67	0.87	0.67
HLA-B*08:02	0.97	0.47	0.97	0.48	HLA-C*07:01	0.85	0.65	0.86	0.65
HLA-B*08:03	0.93	0.37	0.95	0.39	HLA-C*07:02	0.79	0.51	0.76	0.46
HLA-B*14:01	0.71	0.30	0.71	0.31	HLA-C*08:02	0.57	0.20	0.54	0.15
HLA-B*14:02	0.81	0.41	0.79	0.33	HLA-C*12:03	0.67	0.29	0.66	0.27
HLA-B*15:01	0.90	0.66	0.90	0.66	HLA-C*14:02	0.53	0.09	0.52	0.07
HLA-B*15:02	0.79	0.56	0.79	0.55	HLA-C*15:02	0.78	0.50	0.77	0.49
HLA-B*15:03	0.87	0.69	0.88	0.69					

Table S4. 5-fold cross validation results on BD2009 (cv_sr)

Allele	IC_{50} AUC	IC_{50} SRCC	Binary AUC	Binary SRCC	Allele	IC_{50} AUC	IC_{50} SRCC	Binary AUC	Binary SRCC
HLA-A*01:01	0.94	0.52	0.94	0.50	HLA-B*07:02	0.94	0.61	0.94	0.61
HLA-A*02:01	0.95	0.78	0.95	0.78	HLA-B*08:01	0.88	0.59	0.88	0.59
HLA-A*02:02	0.93	0.85	0.93	0.85	HLA-B*08:02	0.93	0.43	0.93	0.45
HLA-A*02:03	0.94	0.80	0.94	0.80	HLA-B*08:03	0.86	0.35	0.88	0.42
HLA-A*02:05	1.00	0.60	1.00	0.61	HLA-B*14:02	0.00	-0.50	0.50	0.50
HLA-A*02:06	0.90	0.78	0.90	0.78	HLA-B*15:01	0.89	0.63	0.89	0.63
HLA-A*02:07	0.85	0.69	0.83	0.73	HLA-B*15:02	0.76	0.51	0.76	0.51
HLA-A*02:10	N/A	N/A	N/A	N/A	HLA-B*15:03	0.89	0.64	0.89	0.64
HLA-A*02:11	0.95	0.78	0.95	0.78	HLA-B*15:09	0.94	0.45	0.91	0.35
HLA-A*02:12	0.97	0.75	0.98	0.76	HLA-B*15:17	0.94	0.74	0.94	0.74
HLA-A*02:16	0.97	0.67	0.98	0.68	HLA-B*18:01	0.90	0.53	0.90	0.50
HLA-A*02:19	0.96	0.63	0.96	0.63	HLA-B*27:01	N/A	N/A	N/A	N/A
HLA-A*02:50	0.99	0.86	0.99	0.87	HLA-B*27:02	N/A	N/A	N/A	N/A
HLA-A*03:01	0.93	0.68	0.93	0.68	HLA-B*27:03	0.96	0.05	0.90	0.05
HLA-A*03:02	N/A	-1.00	N/A	-1.00	HLA-B*27:05	0.94	0.60	0.93	0.59
HLA-A*11:01	0.94	0.76	0.94	0.76	HLA-B*35:01	0.89	0.63	0.89	0.63
HLA-A*23:01	0.92	0.65	0.91	0.63	HLA-B*35:03	N/A	nan	N/A	nan
HLA-A*24:02	0.90	0.59	0.90	0.60	HLA-B*38:01	0.84	0.48	0.84	0.48
HLA-A*24:03	0.97	0.65	0.97	0.64	HLA-B*39:01	0.90	0.63	0.90	0.63
HLA-A*25:01	0.96	0.57	0.96	0.57	HLA-B*40:01	0.96	0.55	0.96	0.52
HLA-A*26:01	0.92	0.46	0.92	0.46	HLA-B*40:02	0.88	0.72	0.88	0.69
HLA-A*26:02	0.97	0.81	0.97	0.80	HLA-B*42:01	N/A	-1.00	N/A	-1.00
HLA-A*26:03	0.96	0.55	0.97	0.56	HLA-B*44:02	0.92	0.53	0.93	0.48
HLA-A*29:02	0.87	0.62	0.86	0.60	HLA-B*44:03	0.92	0.77	0.92	0.77
HLA-A*30:01	0.91	0.69	0.91	0.69	HLA-B*45:01	0.91	0.68	0.90	0.61
HLA-A*30:02	0.82	0.63	0.81	0.61	HLA-B*46:01	0.90	0.45	0.90	0.44
HLA-A*31:01	0.93	0.71	0.93	0.70	HLA-B*48:01	0.95	0.50	0.95	0.50
HLA-A*32:01	0.82	0.65	0.83	0.66	HLA-B*51:01	0.92	0.60	0.92	0.59
HLA-A*33:01	0.90	0.67	0.90	0.66	HLA-B*53:01	0.89	0.77	0.89	0.77
HLA-A*66:01	N/A	0.20	N/A	0.40	HLA-B*54:01	0.90	0.71	0.90	0.70
HLA-A*68:01	0.88	0.76	0.88	0.76	HLA-B*57:01	0.94	0.55	0.94	0.53
HLA-A*68:02	0.90	0.67	0.91	0.67	HLA-B*58:01	0.94	0.59	0.94	0.59
HLA-A*69:01	0.92	0.53	0.93	0.54	HLA-B*58:02	0.49	0.15	0.54	0.18
HLA-A*80:01	0.95	0.66	0.95	0.66	HLA-B*73:01	0.70	0.34	0.67	0.38

Table S5. Standard deviation values of two 5-fold cross validations on BD2013

Test	Alleles	Seq. count	IC50 AUC	IC50 SRCC	Binary AUC	Binary SRCC
All	All	121787	0.15	0.32	0.15	0.32
	HLA-A	72,618	0.10	0.14	0.11	0.14
	HLA-B	46,915	0.18	0.40	0.18	0.40
	HLA-C	2,254	0.13	0.26	0.12	0.27
CD-HIT filtered	All	104,449	0.12	0.21	0.14	0.22
	HLA-A	60,987	0.12	0.16	0.12	0.17
	HLA-B	41,360	0.10	0.21	0.13	0.22
	HLA-C	2,102	0.16	0.27	0.17	0.29

Table S6. Leave-one-allele-out result on BD2013 training dataset

	IC_{50}		Binary			IC_{50}		Binary	
Allele	AUC	SRCC	AUC	SRCC	Allele	AUC	SRCC	AUC	SRCC
HLA-A*01:01	0.77	0.40	0.77	0.37	HLA-B*15:09	0.56	0.11	0.57	0.12
HLA-A*02:01	0.93	0.77	0.93	0.77	HLA-B*15:17	0.79	0.45	0.86	0.57
HLA-A*02:02	0.93	0.85	0.93	0.85	HLA-B*15:42	0.69	0.04	0.68	0.04
HLA-A*02:03	0.94	0.80	0.94	0.80	HLA-B*18:01	0.49	0.09	0.49	0.07
HLA-A*02:04	N/A	N/A	N/A	N/A	HLA-B*27:01	N/A	-1.00	N/A	-1.00
HLA-A*02:05	1.00	0.80	1.00	0.79	HLA-B*27:02	N/A	0.20	N/A	0.20
HLA-A*02:06	0.89	0.76	0.88	0.74	HLA-B*27:03	N/A	0.06	N/A	0.06
HLA-A*02:07	0.83	0.69	0.84	0.73	HLA-B*27:04	N/A	1.00	N/A	0.50
HLA-A*02:10	N/A	N/A	N/A	N/A	HLA-B*27:05	0.64	0.24	0.63	0.18
HLA-A*02:11	0.96	0.82	0.96	0.81	HLA-B*27:06	N/A	-1.00	N/A	1.00
HLA-A*02:12	0.97	0.77	0.97	0.76	HLA-B*27:10	N/A	N/A	N/A	N/A
HLA-A*02:16	0.97	0.68	0.97	0.68	HLA-B*27:20	0.63	0.56	0.68	0.59
HLA-A*02:17	0.58	0.28	0.58	0.28	HLA-B*35:01	0.83	0.59	0.84	0.59
HLA-A*02:19	0.87	0.52	0.87	0.52	HLA-B*35:03	0.87	0.46	0.88	0.48
HLA-A*02:50	0.94	0.78	0.94	0.78	HLA-B*37:01	0.88	0.50	0.87	0.45
HLA-A*03:01	0.91	0.69	0.91	0.69	HLA-B*38:01	0.86	0.62	0.84	0.58
HLA-A*03:02	0.64	0.57	0.61	0.54	HLA-B*39:01	0.86	0.51	0.86	0.51
HLA-A*03:19	0.83	0.52	0.82	0.51	HLA-B*40:01	0.94	0.58	0.94	0.56
HLA-A*11:01	0.89	0.66	0.89	0.67	HLA-B*40:02	0.87	0.65	0.87	0.65
HLA-A*11:02	1.00	0.77	0.97	0.76	HLA-B*40:13	0.58	0.32	0.64	0.42
HLA-A*23:01	0.92	0.69	0.91	0.66	HLA-B*42:01	0.93	0.80	0.94	0.81
HLA-A*24:02	0.75	0.42	0.75	0.43	HLA-B*42:02	0.71	0.50	0.73	0.49
HLA-A*24:03	0.85	0.52	0.85	0.50	HLA-B*44:02	0.93	0.58	0.93	0.55
HLA-A*25:01	0.99	0.47	0.99	0.47	HLA-B*44:03	0.93	0.79	0.93	0.79
HLA-A*26:01	0.91	0.47	0.91	0.46	HLA-B*45:01	0.87	0.60	0.84	0.55
HLA-A*26:02	0.96	0.73	0.96	0.74	HLA-B*45:06	0.88	0.23	0.89	0.23
HLA-A*26:03	0.90	0.48	0.92	0.51	HLA-B*46:01	0.91	0.43	0.91	0.43
HLA-A*29:02	0.75	0.46	0.75	0.43	HLA-B*48:01	0.89	0.47	0.89	0.47
HLA-A*30:01	0.72	0.41	0.74	0.42	HLA-B*51:01	0.83	0.45	0.85	0.45
HLA-A*30:02	0.61	0.21	0.62	0.21	HLA-B*52:01	0.58	0.32	0.58	0.32
HLA-A*31:01	0.77	0.51	0.76	0.49	HLA-B*53:01	0.82	0.61	0.83	0.63
HLA-A*32:01	0.71	0.43	0.72	0.46	HLA-B*54:01	0.76	0.46	0.73	0.41
HLA-A*32:07	0.76	0.36	0.75	0.36	HLA-B*57:01	0.83	0.45	0.83	0.44
HLA-A*32:15	0.64	0.34	0.63	0.32	HLA-B*57:02	0.80	0.71	0.80	0.72
HLA-A*33:01	0.89	0.69	0.88	0.66	HLA-B*57:03	0.88	0.66	0.88	0.68
HLA-A*66:01	0.80	0.34	0.77	0.30	HLA-B*58:01	0.78	0.44	0.78	0.43
HLA-A*68:01	0.74	0.44	0.75	0.45	HLA-B*58:02	0.72	0.53	0.74	0.58
HLA-A*68:02	0.78	0.50	0.78	0.50	HLA-B*73:01	0.69	0.28	0.63	0.20
HLA-A*68:23	0.81	0.56	0.81	0.55	HLA-B*81:01	0.83	0.58	0.87	0.62
HLA-A*69:01	0.91	0.48	0.92	0.50	HLA-B*83:01	0.81	0.34	0.83	0.36
HLA-A*74:01	0.85	0.63	0.80	0.52	HLA-C*03:03	0.67	0.34	0.68	0.35
HLA-A*80:01	0.77	0.31	0.81	0.38	HLA-C*04:01	0.39	-0.06	0.45	-0.10
HLA-B*07:02	0.77	0.46	0.77	0.47	HLA-C*05:01	0.67	0.40	0.66	0.37
HLA-B*08:01	0.80	0.51	0.80	0.50	HLA-C*06:02	0.84	0.66	0.84	0.66
HLA-B*08:02	0.93	0.44	0.93	0.44	HLA-C*07:01	0.72	0.41	0.73	0.41
HLA-B*08:03	0.80	0.24	0.84	0.27	HLA-C*07:02	0.73	0.36	0.71	0.34
HLA-B*14:01	0.72	0.38	0.72	0.40	HLA-C*08:02	0.53	0.04	0.53	0.04
HLA-B*14:02	0.73	0.34	0.75	0.31	HLA-C*12:03	0.65	0.10	0.64	0.09
HLA-B*15:01	0.82	0.54	0.83	0.54	HLA-C*14:02	0.57	0.06	0.58	0.06
HLA-B*15:02	0.73	0.52	0.74	0.53	HLA-C*15:02	0.61	0.19	0.61	0.19
HLA-B*15:03	0.85	0.62	0.85	0.62					

Table S7. Evaluation results of Kim's DCNN and DeepSeqPan

MHC	IEDB Ref	Measure Type	Count	AUC	
				Kim	DeepSeqPan (Binary)
HLA-A*01-01	1028282	t1/2	6	1.00	1.00
HLA-A*02-01	1026371	t1/2	34	0.70	0.76
HLA-A*02-01	1026840	Binary	341	0.85	0.85
HLA-A*02-01	1026840	IC_{50}	22	0.79	0.55
HLA-A*02-01	1026840	t1/2	22	0.69	0.58
HLA-A*02-01	1027079	Binary	15	0.80	0.80
HLA-A*02-01	1027471	Binary	43	0.79	0.88
HLA-A*02-01	1027588	Binary	18	0.70	0.82
HLA-A*02-01	1028285	t1/2	135	0.75	0.72
HLA-A*02-01	1028553	IC_{50}	22	0.85	0.95
HLA-A*02-01	1028554	IC_{50}	44	0.75	0.91
HLA-A*02-01	1028928	Binary	11	0.92	0.94
HLA-A*02-01	1029824	Binary	77	0.59	0.58
HLA-A*03-01	1028288	t1/2	221	0.84	0.85
HLA-A*03-01	1031253	IC_{50}	14	0.96	1.00
HLA-A*11-01	1026891	Binary	16	0.71	0.67
HLA-A*11-01	1028287	t1/2	219	0.79	0.76
HLA-A*24-02	1026840	Binary	346	0.84	0.83
HLA-A*24-02	1026840	IC_{50}	19	0.62	0.61
HLA-A*24-02	1026891	Binary	19	0.55	0.68
HLA-A*24-02	1028289	t1/2	423	0.73	0.74
HLA-A*30-01	1026840	Binary	347	0.87	0.85
HLA-A*30-02	1026840	Binary	360	0.73	0.72
HLA-A*30-02	1026840	IC_{50}	56	0.51	0.55
HLA-A*30-02	1026840	t1/2	56	0.48	0.49
HLA-A*31-01	315312	Binary	8	0.94	0.88
HLA-A*66-01	315312	Binary	16	0.39	0.14
HLA-A*68-01	1026840	Binary	436	0.86	0.84
HLA-A*68-01	1026840	IC_{50}	35	0.84	0.78
HLA-A*68-01	1026840	t1/2	35	0.42	0.33
HLA-B*07-02	1026371	t1/2	33	0.89	0.91
HLA-B*07-02	1026840	Binary	288	0.86	0.81
HLA-B*07-02	1028291	t1/2	136	0.82	0.82
HLA-B*07-02	1028553	IC_{50}	22	0.84	0.90
HLA-B*07-02	1028554	IC_{50}	52	0.80	0.80
HLA-B*07-02	1028928	Binary	11	1.00	1.00
HLA-B*07-02	1031253	IC_{50}	13	1.00	1.00
HLA-B*15-01	1028293	t1/2	570	0.74	0.62
HLA-B*15-02	1027131	Binary	14	1.00	1.00
HLA-B*27-05	1029125	Binary	21	0.97	0.95
HLA-B*27-05	1031253	IC_{50}	12	0.60	0.63
HLA-B*35-01	1028292	t1/2	363	0.81	0.74
HLA-B*35-01	1028554	IC_{50}	56	0.47	0.58
HLA-B*40-01	1026891	Binary	19	0.83	0.81
HLA-B*40-01	1026897	Binary	15	0.80	0.80
HLA-B*44-03	1028554	IC_{50}	46	0.54	0.80
HLA-B*57-01	1028554	IC_{50}	53	0.87	0.89
HLA-B*57-01	1029061	IC_{50}	17	0.90	0.95
HLA-B*58-01	1026840	Binary	433	0.87	0.85
HLA-B*58-01	1026840	IC_{50}	34	0.77	0.53
HLA-B*58-01	1026840	t1/2	34	0.44	0.58
HLA-B*58-01	1026891	Binary	20	0.66	0.67
HLA-B*58-01	1026897	Binary	22	0.81	0.90
HLA-B*27-05	1031959	Binary	13540	0.60	0.60
